# Bilateral Facial Nerve Palsy in a Patient With Leptospirosis From Panama

**DOI:** 10.7759/cureus.106067

**Published:** 2026-03-29

**Authors:** Cristel M Rodriguez-Vargas, Ana B Arauz

**Affiliations:** 1 Infectious Diseases, Hospital Santo Tomas, Panama, PAN; 2 Internal Medicine, Universidad De Panama, Panama, PAN

**Keywords:** bacterial zoonoses, epidemiology, facial paralysis, leptospirosis, tropical medicine

## Abstract

Leptospirosis is a zoonotic tropical disease with worldwide distribution caused by bacteria from the genus *Leptospira*. The clinical picture is assorted, from asymptomatic subclinical disease to more severe forms affecting several organs, including lungs, liver, and kidneys. Neurologic manifestations account for 10%-20% of the cases. The most common neurological presentation is aseptic meningitis, but other syndromes, such as cranial nerve palsies, and encephalitis, are also seen. To our knowledge, this is the first reported case of this type of neurological presentation of leptospirosis in Panama. We present an unusual manifestation of leptospirosis in a 43-year-old man from Panama City who presented with constitutional symptoms and developed bilateral facial palsy and liver involvement during his hospitalization.

## Introduction

Leptospirosis is an infectious disease caused by spirochetes of the genus *Leptospira*. It is a zoonotic waterborne, emerging and re-emerging neglected tropical disease, widely spread among humans and animals all around the world [1,2]. Pathogenic species are responsible for human infections, often occurring after heavy rainfall and flooding [2] but also associated with exposure to environmental sources such as animal urine and other infected animal tissue [3]. Bacteria are shed in urine from animals such as felines, rats, and cattle, which are then transmitted to humans through the exposure of mucosa or breaks in the skin. Human leptospirosis can present with diverse clinical manifestations, ranging from a self-limiting febrile illness to a potentially lethal disease with septic shock and multiple organ failure. The clinical picture is often the same as other tropical diseases, such as malaria, dengue fever, rickettsia infections, and typhoid fever. The classical presentation consists of fever, conjunctival suffusion, hemorrhagic manifestations (nosebleeds, petechiae, blood-tinged urine/stool, and cough-up blood (hemoptysis)), jaundice with pulmonary infiltrates, elevated bilirubin, and renal failure, which are seen two to 30 days after the exposure [4]. Neurological symptoms in relation to leptospirosis, although not the commonest presentation, have been reported over the years, with protean manifestations. Neurologic compromise due to leptospirosis is thought to represent 10%-20% of the cases, and the most common presentation is aseptic meningitis [3-10]. Primary neuroleptospirosis is uncommon and other reported neurological manifestations include stroke, cerebral venous thrombosis, arteritis, subarachnoid hemorrhage, uveitis with blindness, optic neuritis, transverse myelitis, cranial nerve palsy, Guillain-Barré Syndrome, mononeuritis multiplex, peripheral nerve palsy, psychosis, cerebellitis, and chronic meningitis [11]. The prognosis is unknown with a reported mortality of 5%-15%. Some cases resolve spontaneously, while minor cases resolve with oral antibiotics such as doxyxycline. Severe cases are treated with penicillin G, cefotaxime, or ceftriaxone. Early antibiotic therapy is crucial to avoid complications [12]. Other therapies such as corticosteroids and plasmapheresis have been proposed for the treatment of severe presentations of leptospirosis, in lieu of the the role of the immune system in the pathophysiology and manifestations. Corticosteroids were beneficial in some studies; however, there is no robust evidence to recommend this approach in severe cases of leptospirosis [13]. The major benefit appears to be in patients with pulmonary involvement [14,15]. Leptospirosis should be one of many differential diagnoses in endemic regions, considering the appropriate risks of exposure and clinical picture. Empirical therapy should not be withheld while waiting for the confirmatory tests, since some cases can be life-threatening. This case is an example of an unusual presentation of leptospirosis in a patient from Panama.

## Case presentation

Clinical vignette

A 43-year-old man sought medical care in August 2020 (rainy season) from a medical center in Panama City. He complained of being ill for six days, with a 40°C fever, myalgias, and neck pain. He was treated for pharyngitis with azithromycin, acetaminophen, and ibuprofen, which led to improvement of the neck pain, but the fever persisted. Three days before his admission, he noticed an erythematous rash on his upper and lower extremities and had dry cough and sore throat. On his initial physical exam, his vitals were: blood pressure 120/83 mmHg, respiratory rate 111 rpm, and heart rate 25 bpm. He looked ill and jaundiced, with bilateral eye suffusion and rhinorrhea. No rales or other abnormal pulmonary sounds, no heart murmur, or added sounds were found. Neck, axillary, and inguinal adenopathies were absent, and he had no liver or splenic enlargement.

He lives in a suburban location in Panama City, works from home for a call center, and has not traveled outside the country or the countryside since the COVID-19 pandemic isolation began in March 2020. He has two dogs, and one of them is four months old and lacks complete vaccination. He denies recent flooding around his house.

His laboratory exams showed neutrophilia without leukocytosis, lymphopenia, and decreased monocyte count. Hypokalemia, mild elevation of liver enzymes, hyperbilirubinemia, and elevated low-density lactate dehydrogenase (LDH) were also found. Renal function was preserved. Among the acute-phase reactants, C-reactive protein was elevated, but procalcitonin and creatinine kinase (CK) were normal. The urinalysis showed sterile pyuria (see Table 1).

**Table 1:** Laboratory Exams

Laboratory Parameter	Results	Normal Range
White blood cell count	9.8x10^3^/µL	4.5-12.5x10^3^/µL cells
Hemoglobin	16.2 g/dL	13-16 g/dL
Hematocrit	47.8%	36-51%
Platelets	175x10^3^/µL	140-400x10^3^/µL
Neutrophils	92.2%	45-74%
Lymphocytes	2.9%	16-45%
Monocytes	3.1%	4-10%
Basophils	0.3%	0-2%
Eosinophils	1.5%	0-7%
C-reactive protein	241.28 mg/L	0.2-7.48 mg/dL
Procalcitonin	1.03	Less than 1
Creatinine	1.1 mg/dL	0.6-1.3 mg/dL
Blood urea nitrogen (BUN)	10.8 mg/dL	7-18 mg/dL
Sodium	137.2 mmol/L	135-145 mmol/L
Potassium	3.35 mmol/L	3.2-5 mmol/L
Cl	101.8 mmol/L	98-110 mmol/L
Aspartate aminotransferase (AST)	90 IU/L	10-42 UI/L
Alanine aminotransferase (ALT)	137 IU/L	10-40 UI/L
Total bilirubin	3.68 mg/dL	0.2-1 mg/dL
Direct bilirubin	1.15 mg/dL	0-0.2 mg/dL
Indirect bilirubin	2.5 mg/dL	0.2-0.8 mg/dL
Lactate dehydrogenase	223 IU/L	91-180 IU/L
Creatinine kinase	118 IU/L	38-174

The chest X-ray was normal upon admission. An abdominal ultrasound was made because of the elevated liver enzymes, hyperbilirubinemia, and high LDH, which only revealed a mildly enlarged liver due to inflammation. He was started on cefotaxime as empiric therapy for enteric fever/billiary tree-related sepsis as per the Infectious Diseases Society of America (IDSA) guidelines [16]. 

**Figure 1:**
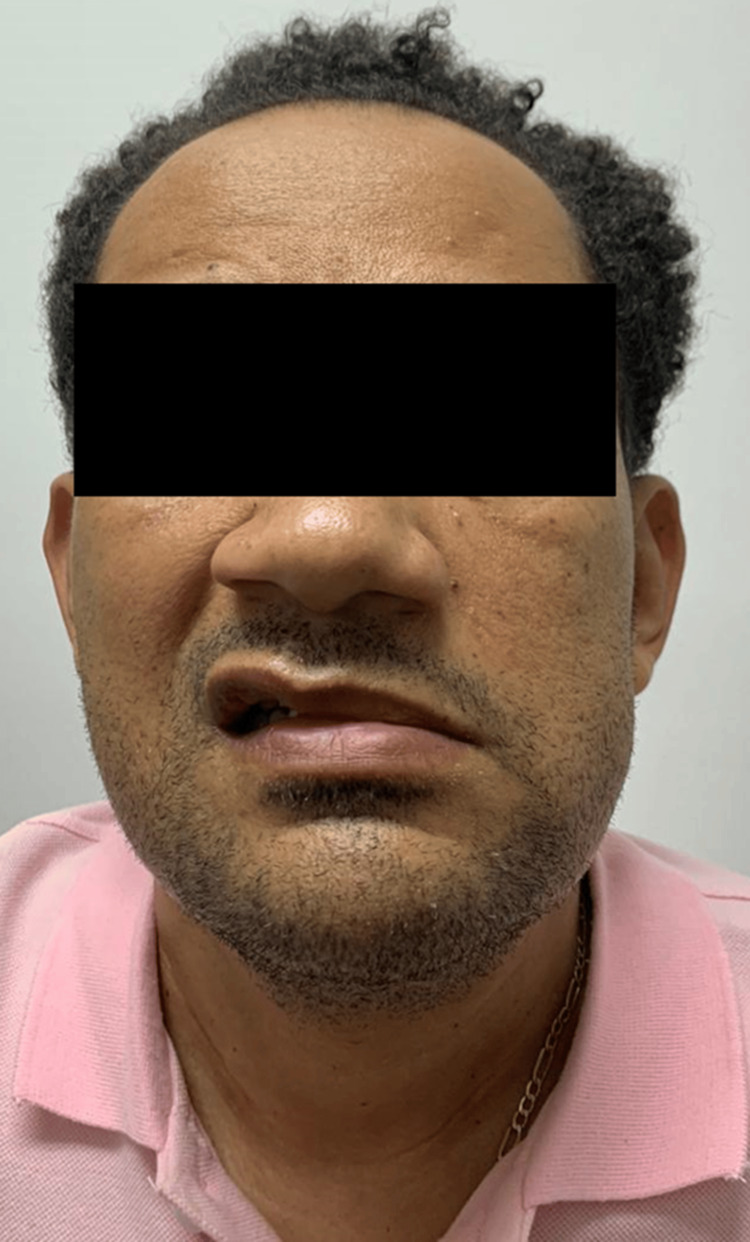
Left facial nerve peripheral palsy Left peripheral facial nerve palsy after three days of admission.

On the third day after his admission, he developed peripheral left facial nerve palsy without other findings on neurological examination (Figure 1). A head CT was performed and was normal. A brain MRI was subsequently obtained and was also unremarkable. By this time, he remained febrile despite antibiotic therapy. Given the presence of focal neurological involvement suggesting possible central nervous system involvement, a lumbar puncture was performed to evaluate for meningeal inflammation and aid in the diagnostic workup; no contraindications were identified. The cerebrospinal fluid (CSF) analysis showed 138 erythrocytes and 38 leukocytes, with 92% monocytes and 8% neutrophils. Chemical analysis revealed protein of 71 mg/dL, glucose of 56 mg/dL, and LDH of 18 U/L. CSF Gram stain was negative. A FilmArray panel of the CSF was also negative for common bacterial, viral, and fungal pathogens causing aseptic meningitis, including herpes simplex virus (HSV)-1 and HSV-2. Because of the erythrocyte count and monocyte-predominant pleocytosis, he was started on acyclovir for suspected herpes simplex encephalitis (sixth day after admission, see Table 2). Liver enzymes and bilirubin levels increased compared to his initial laboratory values. Doxycycline was added to the initial antibiotic regimen (sixth day after admission), targeting possible rickettsial disease.

**Table 2:** Cerebrospinal Fluid Analysis PCR: polymerase chain reaction, LDH: lactate dehydrogenase; CSF: cerebrospinal fluid.

Parameter	Result	Normal range
Color	Transparent	Transparent
Leucocytes	38 cells/mm^3^	0-5 cells/mm^3^
Segmented leucocytes	8%	-
Monocytes	92%	-
Erythrocytes	138 cells/mm^3^	0
Cryptococcal antigen	Negative	Negative
Gram stain	No microorganisms observed	Negative
Acid-fast stain	Negative	Negative
Mycobacterium tuberculosis real-time PCR	Not detected	Not detected
CSF multiplex PCR (*Escherichia coli*, *Hemophilus influenzae*, *Listeria monocytogenes*, *Neisseria meningitidis*, *Streptococcus agalactiae*, *Streptococcus pneumoniae*, cytomegalovirus, enterovirus, herpes simplex virus 1,2,6, parechovirus, varicella zoster virus, *Cryptococcus neoformans*)	Not detected	Not detected
CSF culture	Negative	Negative
Proteins	71 mg/dL	35-45 mg/dL
Glucose	56 mg/dL	Serum glucose 106. CSF to Serum glucose ratio 0.56. Abnormal ratio is considered under 0.4
LDH	18 IU/L	0-40 IU/L

On the seventh day after hospital admission, he developed bilateral peripheral facial nerve palsy and fever. Studies for dengue fever, hepatitis A, B, and C virus (HAV, HBV, and HCV), COVID-19, HIV, syphilis, cytomegalovirus (CMV), and Epstein-Barr virus (EBV) were negative. IgM for leptospirosis returned positive on the 12th day after admission.

The diagnosis of leptospirosis was established through serological testing. An IgM enzyme-linked immunosorbent assay (ELISA), accredited under international standards (code OGA-LE-68-16, granted by the Guatemalan Accreditation Office), was used for the qualitative detection of specific IgM antibodies against *Leptospira antigens*.

Subsequently, the microscopic agglutination test (MAT), a quantitative assay for the titration of serum antibodies against different *Leptospira* serovars, was performed using both the initial serum sample and a second sample. In this case, the convalescent serum sample was obtained three days after the initial sample, demonstrating a threefold increase in antibody titers. Although this interval is shorter than standard recommendations, the observed rise in titers, in conjunction with the clinical context, was considered indicative of a positive result.

Both the IgM ELISA and MAT were performed at the Central Reference Laboratory of the Gorgas Memorial Institute for Health Studies [17].

The patient had a favorable clinical course and received cefotaxime with dose adjustment for central nervous system (CNS) infection for 14 days. He was discharged afebrile, with a marked improvement of constitutional symptoms and liver profile. He was referred for physiotherapy for the facial nerve palsy.

## Discussion

Leptospirosis presents with a wide range of clinical manifestations, from a mild, self-limiting acute febrile illness to a severe, life-threatening condition involving multiple organ failure [4,18]. Neurologic manifestations emerge during the immune phase of the illness, and can involve both the central and peripheral nervous systems [3,7,11,19].

In the present case, the diagnosis was supported by both clinical and epidemiological factors. The patient reported exposure to potential reservoirs, including rodents and an unvaccinated dog, and the illness occurred during the rainy season. These exposures are well-recognized sources of infection through contact with contaminated environments [1,4,18]. Additionally, the case is consistent with national epidemiological patterns reported in Panama, where urban cases and rodent exposure play a significant role in transmission [20].

A notable feature of this case was the development of bilateral facial nerve palsy, an uncommon neurological manifestation of leptospirosis. Although neurological involvement has been described, it is most frequently limited to aseptic meningitis, with cranial neuropathies reported less commonly [3,5,8]. Bilateral facial palsy, in particular, is rare and has been described mainly during the immune phase of the disease [3,5,9]. The temporal progression of symptoms in our patient supports this association.

The underlying mechanism of cranial nerve involvement remains unclear, but it has been hypothesized to result from immune-mediated processes, including vasculitis and circulating immune complexes [5,9]. In this case, CSF findings were consistent with an inflammatory process, supporting this hypothesis.

The differential diagnosis of bilateral facial nerve palsy is broad and includes several infectious diseases [3,6]. In our patient, these causes were systematically excluded through clinical evaluation and complementary studies. Serologic tests and autolimitation of the disease further supported the diagnosis of leptospirosis.

Diagnosis in this case relied on serological methods. Although MAT is considered the reference standard [4], its interpretation can be challenging when samples are obtained within a short interval. In our patient, a threefold rise in antibody titers was observed over a three-day period. While this interval is shorter than standard recommendations, the increase in titers, together with the clinical presentation, was considered sufficient to support the diagnosis.

Early initiation of antibiotic therapy is recommended in suspected leptospirosis [4,12]. In this case, empirical treatment with cefotaxime and doxycycline was initiated due to a broad differential diagnosis. Once leptospirosis was confirmed, doxycycline was discontinued and cefotaxime was continued at a non-meningitis dose, with a favorable clinical outcome.

This case illustrates an atypical neurological presentation of leptospirosis and underscores the importance of considering this diagnosis in patients presenting with bilateral facial nerve palsy in endemic settings such as Panama. Early recognition, appropriate diagnostic testing, and timely treatment are essential to improve outcomes.

## Conclusions

This case highlights bilateral facial nerve palsy as a rare neurological manifestation of leptospirosis, likely occurring during the immune phase of the disease. Leptospirosis should be considered even in atypical presentations, particularly in the presence of relevant environmental and animal exposures. Maintaining a high index of suspicion is essential to enable early diagnosis and timely treatment, thereby improving clinical outcomes.
